# Laryngeal mask airway insertion by classic and thumb insertion technique: a comparison

**DOI:** 10.12688/f1000research.2-123.v1

**Published:** 2013-05-09

**Authors:** Monica Goyal, Akanksha Dutt, Anjum S Khan Joad

**Affiliations:** 1 Department of Anesthesiology, O.P. Jindal Hospital, Hisar, 125005, India; 2 Department of Anesthesiology, Sawai ManSingh Medical College, Jaipur, 302004, India; 3 Department of Anesthesiology, Bhagawan Mahavir Medical Research Center, Jaipur, 302017, India

## Abstract

We evaluated the efficacy of an alternative technique, for insertion of the silicone laryngeal mask airway (LMA) Classic™ in 40 American Society of Anesthesiologists grade ASA I and II patients scheduled for elective surgery. In group I (Index Finger group), the LMA was inserted by the classic index finger technique and, in group T (Thumb Insertion group), the thumb insertion technique was used. Ease of insertion, fiberoptic laryngoscopic position, cuff pressures and laryngopharyngeal morbidity were assessed in both study groups. On statistical analysis, both groups were comparable in all respects. From our study it can be concluded that thumb insertion is an effective insertion technique for the LMA Classic™.

## Introduction

The thumb insertion technique offers an attractive alternative technique for insertion of a laryngeal mask airway (LMA). An LMA is conventionally inserted by the index finger insertion technique as described by Dr. Archie Brain ^1, 2^. However, there are certain conditions where LMA insertion using the index finger is difficult, as it is not possible to reach the head end of the patient, for instance during mass casualty (e.g. fire, building collapse, etc.) or in case of a patient with a stereo tactic frame ^3^. Also, paramedical workers are often less comfortable working at the head end of the patient. In such circumstances, an alternative technique of insertion may be useful. Therefore, this study was planned to assess the thumb insertion technique.

This study was performed using the silicone LMA (LMA Classic™) to compare the two insertion techniques with respect to ease of insertion, fiberoptic laryngoscopic position, cuff pressure and patient comfort in patients for elective general anesthesia.

## Material and methods

After approval from the hospital research and ethical committee, a prospective, randomized double-blind study was conducted on patients of American Society of Anesthesiologists ASA grade I & II between 18–60 years of age, undergoing elective surgery.

Exclusion criteria were as follows ^1^:

1. Risk of aspiration (full stomach, gastroesophageal reflux disease, pregnancy).

2. Mouth opening < 2.5 cm.

3. Weight < 40 kg or > 110 kg.

4. Respiratory tract pathology.

5. Cervical spine disease.

After informed consent was obtained, patients were randomly allocated into two groups of 20 each. In group Index (I), the silicone LMA was inserted using the index finger insertion technique and in group Thumb (T), the silicone LMA was inserted using the thumb insertion technique. Randomization was done by opening sealed numbered envelopes.

Patients were premedicated with oral diazepam 0.1 mg/kg two hours before anesthesia and intravenous Ondansetron 0.01 mg/kg just before induction of anesthesia. Intra-operative monitoring included continuous electrocardiogram (ECG), non-invasive blood pressure (NIBP), oxygen saturation (SpO _2_) end tidal CO _2_ (Et CO _2_) and airway pressure.

Following preoxygenation, anesthesia was induced with intravenous fentanyl 2 mcg/kg. Two minutes after administering fentanyl, propofol 2.5 mg/kg was given intravenously ^4^. One minute after administration of propofol, LMA insertion was attempted. During LMA insertion, if the mouth opening was inadequate, or if purposeful movement in patients was observed, further bolus doses of 10 mg propofol were given to facilitate insertion. In all patients the LMA was inserted by an experienced anesthesiologist who was well versed with both techniques of LMA insertion.

In group Index (I), the LMA was inserted from the head end of the patient after partially inflating the cuff ^5^ (i.e. filled with half the recommended air in the cuff), and lubricating the posterior surface of the cuff with water-soluble jelly. The patient's head was supported on a firm ring with neck flexed and head extended. The tube portion of the laryngeal mask was grasped as if it were a pen; the index finger was pressed on the point where the tube adjoins the mask. The patient’s mouth was opened; the tip of the mask was placed against the inner surface of the upper incisors or gums with the aperture facing anteriorly (and the black line facing the patient's upper lip). The mask was pressed back against the hard palate to keep it flattened as it advanced into the hypopharynx, using the index finger to push upward against the palate. The tube was grasped with the other hand, straightened slightly, and then pressed down with a single, quick but gentle movement until a definite resistance was felt.

In group T, the LMA was inserted from the right side of the patient ^6, 7^, i.e. the operator stood facing the patient, in the angle made by the chest and right arm of the patient. After partially inflating the cuff (i.e. filled with half the recommended air in the cuff), the posterior surface of the cuff was lubricated with water-soluble jelly. The patient's head was supported on a firm ring with neck flexed and head extended. The tube portion of the laryngeal mask was grasped in a pen-like fashion; the thumb (instead of the index finger) was pressed on the point where the tube adjoins the mask. After opening the patient’s mouth, the tip of the mask was placed against the inner surface of the upper incisors or gums with the aperture facing anteriorly (and the black line facing the patient’s upper incisors). The mask was pressed back against the hard palate to keep it flattened as it advanced into the hypopharynx until a definite resistance was felt. In this technique, the thumb was used to apply pressure against the hard palate while advancing the LMA. The tube was grasped with the other hand while removing the thumb ^6^.

After insertion, cuff inflation of either device was to a "just seal" pressure or up to a maximum of 60 cm H _2_O, as measured with a simple hand-held aneroid manometer ^8^. The volume of air used was recorded, and a larger device was substituted if leaks persisted on gentle manual ventilation.

Insertion success was assessed by the following criteria:

1. Establishing a clear airway.

2. Rising up of device during cuff inflation.

3. Anterior neck filling with device inflation.

4. The device remained in midline with the black line on the posterior side of airway tube remaining in midline in line with the upper incisors.

Anesthesia was maintained with isoflurane and nitrous oxide in oxygen and vecuronium bromide (0.08 mg/kg initially followed by 0.01 mg/kg every 30 minutes). Mechanical ventilation was volume controlled and time cycled with a tidal volume (5–8 ml/kg) set to maintain peak inspiratory pressure less than 20 cm of H _2_O, and ventilator frequency was adjusted to maintain EtCO _2_ at 30–38 mm of Hg with an I/E ratio of 1:2.

Optimal ventilation was assessed by the following criteria ^9^:

a. Adequate chest expansion.

b. Stable oxygenation.

c. Square wave capnograph.

Soon after this, a fiberoptic bronchoscope was passed through the device and the view was graded as follows ^10, 11^:

a. Vocal cords fully visible.

b. Vocal cords & posterior epiglottis visible.

c. Vocal cords & anterior epiglottis visible.

d. Vocal cords not seen but ventilation adequate.

For our study, fiberoptic view grades a. and b. were labeled good and c. and d. were labeled poor.

After surgery, neuromuscular block was antagonized with neostigmine (0.05 mg/kg) and glycopyrrolate (0.01 mg/kg). LMA was removed after deflating the cuff when the patient regained consciousness and protective airway reflexes. The presence of blood on the LMA cuff was recorded.

In the postoperative period, patients were asked if they had a sore throat in the recovery room and 24 hours postoperatively. The responses were graded as follows.

a. Nil

b. Mild

c. Moderate

d. Severe

Statistical analysis was done using Chi-square test with Yates correction for qualitative analysis and analysis of variance (ANOVA) for quantitative analysis.

## Results

The mean age of first group with index finger insertion was 43.15 years whereas the mean age of second group with index finger insertion technique was 45.70 years. The mean weight of first group was 61.10 and that of second group was 55.3 years. The male: Female ratio in both groups was 1:4. There was no statistically significant difference between the two groups ( Table 1). The time taken, number of attempts, cuff pressure and fiberoptic view scores were comparable in the two groups ( Table 2). The insertion success of LMA & laryngopharyngeal morbidity was statistically comparable between both groups ( Table 3). The time taken and number of attempts for LMA classic insertion were comparable irrespective of the technique used. No significant difference was found in the cuff pressure, fiberoptic view scores and pharyngeal morbidity in both study groups. However, the sample size was small.

**Table 1.** Demographic profile of the patients.

Parameters	Group		P-value	Significance
	Index	Thumb		
Age (Years)	43.15 ± 7.41 SD	45.70 ± 10.86 SD	>0.05	NS
Weight (Kilograms)	61.10 ± 17.44 SD	55.30 ± 09.55 SD	>0.05	NS
Male: Female	1:4	1:4	–	–

Both the study groups were statistically similar with respect to age and weight. NS = Not significant.

**Table 2.** Ease of insertion.

Parameters	Group (Mean + SD of various parameters)		P-value	Significance
	Index	Thumb		
**Time taken for insertion** (Seconds)	29.00 ± 28.60	134.00 ± 17.31	>0.05	NS
**Cuff pressure** (mm of Hg)	43.40 ± 10.79	42.30 ± 10.53	>0.05	NS
**No of attempt**	01.20 ± 000.51	01.25 ± 00.54	>0.05	NS
**Fiber optic view**	01.40 ± 00.58	01.80 ± 00.81	>0.05	NS

The time taken, number of attempts, cuff pressure and fiberoptic view scores were comparable in the two groups. NS = Not significant.

**Table 3.** Insertion success.

Parameter		Group		SD	P-value	Significance
		Index	Thumb			
Clear airway	Yes	20	20	–	–	–
	No	0	0			
Rising up of device	Yes	19	17	0.278	>0.05	NS
	No	1	3			
Anterior neck filling	Yes	18	17	0.000	>0.05	NS
	No	2	3			
Device remained in midline	Yes	20	20	0.000	>0.05	NS
	No	0	0			
Blood	Present	2	4	0.278	>0.05	NS
	Not Present	18	16			

The insertion success of LMA & laryngopharyngeal morbidity was statistically comparable between both groups.

Results of silicone laryngeal mask airway insertion Results of the comparison in insertion technique of a silicone laryngeal mask airway (LMA) into patients using either the 'index finger' technique or the 'thumb' technique.

## Discussion

The manufacturers of the LMA Classic have recommended thumb insertion as one of the methods of insertion ^7^, but there is little evidence in the literature available on its success. In this study, an attempt was made to compare the index finger and thumb insertion techniques for the LMA classic insertion. The users of LMA had a shorter learning curve compared to the endotracheal tube, and paramedical workers lacking advanced airway training easily master the skill of inserting an LMA ^12, 13^. As this group of care givers may not be as comfortable as doctors in working at the head end of the patient, the thumb insertion technique is an attractive option (this technique was demonstrated by Dr. Chandy Varghese at an airway workshop at the All India Institute of Medical Sciences, New Delhi, India in 1999, citing the reluctance of paramedical workers to work at the head end ^14^). Also, in conditions of mass casualty e.g. fire; building collapse or earthquake, when it may not be possible to reach the head end of the patient, the thumb insertion technique would be useful. The time taken for the successful insertion of an airway device when used for airway management during anesthesia or in apneic patients during resuscitation is crucial and, therefore, has to be reasonably brief. In the present study, the time of LMA insertion was defined as the time taken from picking up the device until the time at which positive pressure ventilation was started. Even though in our study, the mean time taken in the thumb insertion group (34.00 + 17.31 sec) was longer than in the index finger group (29.00 + 28.6 sec), the difference was not statistically significant. The number of attempts required for LMA insertion using either technique was also comparable statistically.

Silva and Brimacombe ^15^ (1996) described a non-conventional (thumb) insertion technique of the LMA for general anesthesia during stereotactic implantation of fetal hypophysis in Parkinson’s disease in five patients, as the conventional approach from the patient’s head end was impeded by the stereotactic frame.

The LMA cuff pressures in both study groups were also found to be comparable. A statistically significant difference was not found in the fiberoptic view scores between the two study groups, emphasizing the fact that technique of insertion did not influence correct placement of the LMA and effective ventilation. No significant difference in the incidence of sore throat or blood on device was found in the study groups.

Most of us are accustomed to efficiently placing the LMA by the index finger technique in the operation theatre, but we also need to master the thumb technique to handle difficult situations outside theatre.

## Conclusion

In this patient population, the thumb insertion technique was as effective as index finger insertion technique with respect to ease of insertion and insertion success. It also provides optimal ventilation, comparable fiberoptic view scores and comparable incidence of sore throat. It is important for doctors and paramedical workers to learn both techniques, especially for situations outside the carefully controlled operating theatre environment.
