# Supplementary material for: Laryngeal mask airway insertion by classic and thumb insertion technique: a comparison
Source: F1000Res. 2013 May 9;2:123. [Version 1] doi: 10.12688/f1000research.2-123.v1 (PMC3790608; doi:10.12688/f1000research.2-123.v1)
Supplement: Peer review history

# Peer review: Referee response for version 1

**Published:** 2013-05-14

**Authors:** Brian J Egan

**Affiliations:** 1 Department of Anesthesiology, Columbia University, New York, NY, USA

I have read this submission. I believe that I have an appropriate level of expertise to confirm that it is of an acceptable scientific standard.

# Peer review: Referee response for version 1

**Published:** 2013-05-10

**Authors:** Yehuda Ginosar

**Affiliations:** 1 Department of Anesthesiology and Critical Care Medicine, Hadassah Hebrew University Medical Center, Jerusalem, Israel

This article was well designed, analyzed and presented. It is not “science” but it does address a clinically useful question and does so using an appropriate scientific methodology. The only comments that I would make require minor changes. 

- I suggest modifying the names of the groups to “anesthesiologist at head of patient” and “anesthesiologist facing patient” – or possibly a more elegant phraseology (south-facing vs north-facing approach?) - this is really the main difference between the techniques – whether you use the thumb or index finger is a secondary difference derived from the first.
- This should also appear in the title. 
- In table 3, please add a footnote explaining IS1, IS2 etc and OV 1, OV 2 etc – I know these terms appear in the text but each table should be self-explanatory.

I have read this submission. I believe that I have an appropriate level of expertise to confirm that it is of an acceptable scientific standard.
